# Supplementary material for: Knowledge sharing behaviour among head nurses in online health communities: The moderating role of knowledge self-efficacy
Source: PLoS One. 2023 Jan 19;18(1):e0278721. doi: 10.1371/journal.pone.0278721 (PMC9851523; doi:10.1371/journal.pone.0278721)
Supplement: S1 Appendix — (DOCX) [file pone.0278721.s001.docx]

# Appendix 1

| Scale | Code | Statement | Source(s) |
| --- | --- | --- | --- |
| Trust | TRU1 | We were usually considerate of one another’s feelings in online healthcare communities. | Jarvenpaa and Leidne [1]  Hassandoust et al. [2] |
|  | TRU2 | The people in online healthcare communities were friendly. |  |
|  | TRU3 | I could rely on those with whom I worked in online healthcare communities. |  |
|  | TRU4 | Overall, the people in online health communities were trustworthy. |  |
| Reciprocity | REC1 | When I share knowledge in online healthcare communities, I believe that my questions will be answered in the future. | **Bock et al.** [3]  **Zhang et al.** [4] |
|  | REC2 | I believe that other members whom I interact with would help me whenever I am in need in online healthcare communities. |  |
|  | REC3 | When I share my knowledge in online healthcare communities, I expect the other members to respond whenever I am in need. |  |
| Reputation Scale | REP1 | I earn respect from others by participating in online healthcare communities. | Wasko and Faraj [5]  Zhang et al. [4] |
|  | REP2 | I feel that participation improves my status in online healthcare communities. |  |
|  | REP3 | My participation in online healthcare communities can enhance my reputation in my professional field. |  |
|  | REP4 | I can earn feedback or rewards through participation by representing my reputation and status in the online healthcare communities. |  |
| Ability to Share | ABS1 | I am fully capable of sharing my knowledge with others in online healthcare communities. | Radaelli et al. [6] |
|  | ABS2 | If it depended only on me, I would exhaustively share my knowledge in online healthcare communities. |  |
|  | ABS3 | I am fully capable of articulating my knowledge in written or spoken form in online healthcare communities. |  |
|  | ABS4 | I believe I am fully capable of sharing my knowledge at any time in online healthcare communities. |  |
|  | ABS5 | The knowledge I share with my colleagues would be very useful to them in online healthcare communities. |  |
| Knowledge Self efficacy | KSE1 | My personal expertise will display its value if shared within the online healthcare communities. | Bock and Kim [7]  Lu et al. [8] |
|  | KSE2 | My limited knowledge, even if shared, will generate little effect within the online healthcare communities. |  |
|  | KSE3 | I am confident that my knowledge sharing would improve work processes in online healthcare communities. |  |
|  | KSE4 | I am confident that my knowledge sharing would increase the productivity in the online healthcare communities |  |
| Knowledge Sharing Behaviour | KSB1 | In daily work, I take the initiative to share my work-related knowledge with my colleagues in online healthcare communities. | Bock and Kim [7]  Lu et al. [8] |
|  | KSB2 | I keep my work experience and never share it with others easily in online healthcare communities. |  |
|  | KSB3 | After learning new knowledge useful to work, I promote it to let more people learn it in online healthcare communities. |  |
|  | KSB4 | I actively use online healthcare community sources available to share my knowledge. |  |
|  | KSB5 | So long as the other colleagues need it, I always tell whatever I know without any hoarding in online healthcare communities |  |

**References**

1. Jarvenpaa SL, Leidner DE. Communication and trust in global virtual teams. Organ Sci. 1999;10(6):791–815.

2. Hassandoust F, Logeswaran R, Farzaneh Kazerouni M. Behavioral factors influencing virtual knowledge sharing: theory of reasoned action. J Appl Res High Educ. 2011;3(2):116–34.

3. Bock G-W, Zmud RW, Kim Y-G, Lee J-N. Behavioral intention formation in knowledge sharing: Examining the roles of extrinsic motivators, social-psychological forces, and organisational climate. MIS Q. 2005;29:87–111.

4. Zhang X, Liu S, Deng Z, Chen X. Knowledge sharing motivations in online health communities: A comparative study of health professionals and normal users. Comput Human Behav. 2017;75:797–810.

5. Wasko M, Faraj S. Why should I share? Examining social capital and knowledge contribution in electronic networks of practice. MIS Q. 2005;29(1): 35–57.

6. Radaelli G, Lettieri E, Mura M, Spiller N. Knowledge sharing and innovative work behaviour in healthcare: A micro-level investigation of direct and Indirect effects. Creat Innov Manag. 2014;23(4):400–14.

7. Bock G, Kim Y. Breaking the myths of rewards: An exploratory study of attitudes about knowledge sharing. Info Res Manag Jour. 2001; 15(2):1-8/

8. Lu L, Leung K, Koch PT. Managerial knowledge sharing: The role of individual, interpersonal, and organisational factors. Manag Organ Rev. 2006;2(1):15–41.
