# Supplementary material for: Knowledge sharing behaviour among head nurses in online health communities: The moderating role of knowledge self-efficacy
Source: PLoS One. 2023 Jan 19;18(1):e0278721. doi: 10.1371/journal.pone.0278721 (PMC9851523; doi:10.1371/journal.pone.0278721)
Supplement: Attachment — Submitted filename: PONE-D-SE.pdf [file pone.0278721.s003.pdf]

PLOS ONE

Knowledge Sharing Behaviour among Head Nurses in Online Health Communities: The Moderating Role of Knowledge Self-Efficacy

--Manuscript Draft--

Manuscript Number:	PONE-D-22-23050
Article Type:	Research Article
Full Title:	Knowledge Sharing Behaviour among Head Nurses in Online Health Communities: The Moderating Role of Knowledge Self-Efficacy
Short Title:	Knowledge Sharing Behaviour: The Moderating Role of Knowledge Self-Efficacy
Corresponding Author:	Mu'taman Khalil Mohmoud Jarrar, PhD<br>Imam Abdulrahman Bin Faisal University College of Medicine<br>Dammam, SAUDI ARABIA
Keywords:	Individual factors, knowledge self-efficacy, knowledge sharing behaviour, Online Health Communities, Head nurses, PLS-SEM.
Abstract:	Background: Head nurses are vital in understanding and encouraging knowledge sharing among their followers. However, few empirical studies have highlighted their contribution to knowledge-sharing behaviour in Online Health Communities (OHCs). In addition, scant literature has examined the moderating role of knowledge self-efficacy in this regard.<br>Purposes: This study adopted social cognitive theory to test the moderating role of self-efficacy between four selected individual factors of head nurses (i.e., Trust, Reciprocity, Reputation, and Ability to Share) and their knowledge-sharing behaviour in OHCs in Jordan.<br>Method: The data were obtained by using a self-reported survey from 283 head nurses in 22 private hospitals in Jordan. A structural equation modelling approach (i.e. PLS-SEM) was unitized to evaluate the study's measurement and structural model.<br>Results: Knowledge self-efficacy moderates the relationship between the three individual factors (i.e., Trust, Reciprocity, and Reputation) and knowledge-sharing behaviour s. However, the self-efficacy did not moderate the relationship between the ability to share and knowledge-sharing behaviours.<br>Implications : This study contributes to understanding the moderating role of knowledge self-efficacy among head nurses in online healthcare communities. Moreover, this study provides guidelines for head nurses to become active members in knowledge sharing in OHCs. The findings of this study offer a basis for further research on knowledge sharing in the healthcare sector.
Order of Authors:	Salah Shehab
	Mohammad Al-Bsheish
	Ahmed Meri
	Mohammed Dauwed
	Badr K. Aldhmadi
	Adi Alsyouf
	Khalid Al-Mugheed
	Haitham Mohsin Kareem
	Mu'taman Khalil Mohmoud Jarrar, PhD
Additional Information:	
Question	Response
**Financial Disclosure**<br><br>Enter a financial disclosure statement that	The authors received no specific funding for this work.

Powered by Editorial Manager® and ProduXion Manager® from Aries Systems Corporation

describes the sources of funding for the work included in this submission. Review the submission guidelines for detailed requirements. View published research articles from *PLOS ONE* for specific examples.<br><br>This statement is required for submission and **will appear in the published article** if the submission is accepted. Please make sure it is accurate.<br><br>**Unfunded studies**<br>Enter: *The author(s) received no specific funding for this work.*<br><br>**Funded studies**<br>Enter a statement with the following details:<br>• Initials of the authors who received each award<br>• Grant numbers awarded to each author<br>• The full name of each funder<br>• URL of each funder website<br>• Did the sponsors or funders play any role in the study design, data collection and analysis, decision to publish, or preparation of the manuscript?<br>• **NO** - Include this sentence at the end of your statement: *The funders had no role in study design, data collection and analysis, decision to publish, or preparation of the manuscript.*<br>• **YES** - Specify the role(s) played.<br><br>* typeset	
**Competing Interests**<br><br>Use the instructions below to enter a competing interest statement for this submission. On behalf of all authors, disclose any competing interests that could be perceived to bias this work—acknowledging all financial support and any other relevant financial or non-financial competing interests.<br><br>This statement is required for submission and **will appear in the published article** if the submission is accepted. Please make sure it is accurate and that any funding	The authors have declared that no competing interests exist.

sources listed in your Funding Information later in the submission form are also declared in your Financial Disclosure statement.<br><br>View published research articles from *PLOS ONE* for specific examples.<br><br>**NO authors have competing interests**<br><br>Enter: *The authors have declared that no competing interests exist.*<br><br>**Authors with competing interests**<br><br>Enter competing interest details beginning with this statement:<br><br>*I have read the journal's policy and the authors of this manuscript have the following competing interests: [insert competing interests here]*<br><br>* typeset	
**Ethics Statement**<br><br>Enter an ethics statement for this submission. This statement is required if the study involved:<br><br>• Human participants<br>• Human specimens or tissue<br>• Vertebrate animals or cephalopods<br>• Vertebrate embryos or tissues<br>• Field research<br><br>Write "N/A" if the submission does not require an ethics statement.<br><br>General guidance is provided below. Consult the submission guidelines for detailed instructions. **Make sure that all information entered here is included in the Methods section of the manuscript.**	Ethical approval number UNITEN/COGS 23/2/1/PM20604 was attained from the College of Graduate Studies, Universiti Tenaga Nasional, Malaysia, on 25 April 2018. "Written consent was obtained from all the respondents after they were informed regarding their right to withdraw from participation at any time, that data would be only for academic purposes, and their responses would be confidential".

**Format for specific study types**<br><br>**Human Subject Research (involving human participants and/or tissue)**<br>• Give the name of the institutional review board or ethics committee that approved the study<br>• Include the approval number and/or a statement indicating approval of this research<br>• Indicate the form of consent obtained (written/oral) or the reason that consent was not obtained (e.g. the data were analyzed anonymously)<br><br>**Animal Research (involving vertebrate animals, embryos or tissues)**<br>• Provide the name of the Institutional Animal Care and Use Committee (IACUC) or other relevant ethics board that reviewed the study protocol, and indicate whether they approved this research or granted a formal waiver of ethical approval<br>• Include an approval number if one was obtained<br>• If the study involved *non-human primates*, add *additional details* about animal welfare and steps taken to ameliorate suffering<br>• If anesthesia, euthanasia, or any kind of animal sacrifice is part of the study, include briefly which substances and/or methods were applied<br><br>**Field Research**<br><br>Include the following details if this study involves the collection of plant, animal, or other materials from a natural setting:<br>• Field permit number<br>• Name of the institution or relevant body that granted permission	
**Data Availability**<br><br>Authors are required to make all data underlying the findings described fully available, without restriction, and from the time of publication. PLOS allows rare exceptions to address legal and ethical concerns. See the PLOS Data Policy and FAQ for detailed information.	Yes - all data are fully available without restriction

A Data Availability Statement describing where the data can be found is required at submission. Your answers to this question constitute the Data Availability Statement and **will be published in the article**, if accepted.<br><br>**Important:** Stating 'data available on request from the author' is not sufficient. If your data are only available upon request, select 'No' for the first question and explain your exceptional situation in the text box.<br><br>Do the authors confirm that all data underlying the findings described in their manuscript are fully available without restriction?	
**Describe where the data may be found in full sentences. If you are copying our sample text, replace any instances of XXX with the appropriate details.**<br><br>• If the data are **held or will be held in a public repository**, include URLs, accession numbers or DOIs. If this information will only be available after acceptance, indicate this by ticking the box below. For example: *All XXX files are available from the XXX database (accession number(s) XXX, XXX.).*<br>• If the data are all contained **within the manuscript and/or Supporting Information files**, enter the following: *All relevant data are within the manuscript and its Supporting Information files.*<br>• If neither of these applies but you are able to provide **details of access elsewhere**, with or without limitations, please do so. For example:<br><br>*Data cannot be shared publicly because of [XXX]. Data are available from the XXX Institutional Data Access / Ethics Committee (contact via XXX) for researchers who meet the criteria for access to confidential data.*<br><br>*The data underlying the results presented in the study are available from (include the name of the third party*	The data underlying the results presented in the study are available upon request from the corresponding author.

and contact information or URL).<br>• This text is appropriate if the data are owned by a third party and authors do not have permission to share the data.<br><br>* typeset	
Additional data availability information:	

- knowledge-sharing behaviour may be developed from skills, knowledge, and experiences of the nurses themselves. The more knowledge the nurses have, the more their knowledge-sharing behaviour develop. In addition, the size of the hospital also affects the knowledge of the nurses, for example, a large hospital would conduct effective training sessions for nurses, while a small hospital would not.

- The research findings should be compared with other areas in Jordan.

Powered by Editorial Manager® and ProduXion Manager® from Aries Systems Corporation

Manuscript

Click here to access/download;Manuscript;Knowledge Sharing Behaviour, Clean version.docx

Knowledge Sharing Behaviour among Head Nurses in Online Health Communities: The Moderating Role of Knowledge Self-Efficacy

Salah Shehab*[1], Mohammad Al-Bsheish*[2,3], Ahmed Meri[4], Mohammed Dauwed[5], Haitham Mohsin Kareem[6], Badr K. Aldhmadi[7], Adi Alsyouf[8], Khalid Al-Mugheed[9], Mu'taman Jarrar*[10,11]

[1] College of Graduate Studies, Universiti Tenaga Nasional, Kajang, Selangor, Malaysia, dr-salah-shehab@email.com

[2] Health Management Department, Batterjee Medical College, Jeddah 21442, Saudi Arabia Email: mohammed.ghandour@bmc.edu.sa

[3] Al-Nadeem Governmental Hospital, Ministry of Health, Amman 11118, Jordan Email:gandour1984@yahoo.com gandour1984@yahoo.com

[4] Department of Medical Instrumentation Techniques Engineering, Al-Hussain University College, Al-Hussain University College, 56001 Karbala,, Iraq

[5] Department of Medical Instrumentation Techniques Engineering, Dijlah University College, 10022 Baghdad, Iraq

[6] Department of Accounting, Southern Technical University, 61006 Basrah, Iraq

[7] Department of Health Management, College of Public Health and Health Informatics, University of Ha'il, 81451 Ha'il, Saudi Arabia

[8] Department of Managing Health Services and Hospitals, Faculty of Business Rabigh,College of Business (COB), King Abdulaziz University, Jeddah 21991, Saudi Arabia.

[9] Surgical Nursing Department, Faculty of Nursing, Near East University, Nicosia 99138, Cyprus;khalid.edu@yahoo.com.

[10] Vice Deanship for Quality and Deveopment, Imam Abdulrahman Bin Faisal University, Dammam, Eastern Province, Saudi Arabia, mkjarrar@iau.edu.sa

[11] Medical Education Department, College of Medicine, Imam Abdulrahman Bin Faisal University,Dammam 34212, Saudi Arabia.

***Corresponding Authors**

1. **Salah Shehab**
2. **Mohammad Al-Bsheish**
3. **Mu'taman Jarrar**

Knowledge Sharing Behaviour among Head Nurses in Online Health Communities: The Moderating Role of Knowledge Self-Efficacy

Abstract

Background: Head nurses are vital in understanding and encouraging knowledge sharing among their followers. However, few empirical studies have highlighted their contribution to knowledge-sharing behaviour in Online Health Communities (OHCs). In addition, scant literature has examined the moderating role of knowledge self-efficacy in this regard.

Purposes: This study adopted social cognitive theory to test the moderating role of self-efficacy between four selected individual factors of head nurses (i.e., Trust, Reciprocity, Reputation, and Ability to Share) and their knowledge-sharing behaviour in OHCs in Jordan.

Method: The data were obtained by using a self-reported survey from 283 head nurses in 22 private hospitals in Jordan. A structural equation modelling approach (i.e. PLS-SEM) was unitized to evaluate the study's measurement and structural model.

Results: Knowledge self-efficacy moderates the relationship between the three individual factors (i.e., Trust, Reciprocity, and Reputation) and knowledge-sharing behaviours. However, the self-efficacy did not moderate the relationship between the ability to share and knowledge-sharing behaviours.

Implications: This study contributes to understanding the moderating role of knowledge self-efficacy among head nurses in online healthcare communities. Moreover, this study provides guidelines for head nurses to become active members in knowledge sharing in OHCs. The findings of this study offer a basis for further research on knowledge sharing in the healthcare sector.

Keywords: Individual factors, knowledge self-efficacy, knowledge sharing behaviour, Online Health Communities, Head nurses, PLS-SEM.

1. Introduction

Knowledge-sharing behaviour is becoming increasingly indispensable in today's business environment (Jinyang, 2015; Tran, 2021). Knowledge sharing is an essential resource for effectively implementing essential business functions, and like other industries, healthcare organisations are beginning to use knowledge sharing as a new practice. Knowledge sharing is a conveyance behaviour wherein individuals disperse their knowledge, experiences, and skills to others (Nguyen et al., 2021). Effective knowledge sharing is vital in healthcare organisations because it significantly enhances the quality of care and patient safety (Mura et al., 2016). Healthcare workers can use knowledge sharing for their patients, making it easier to share information about their diagnoses and treatments (Bouma, 2011). Thus, knowledge sharing is a strong element for improvements and further development within the healthcare sector (Kim et al., 2012; Mc Evoy et al., 2019).

The existence of Online Communities (OC) can facilitate knowledge-sharing (Hsu et al., 2007; Mozaffar et al., 2022). In contrast to traditional knowledge sharing in real-world communities, members of OC are distributed across geographic locations. Difficulties related to face-to-face knowledge exchanges among OC members may weaken the bond among OC. Therefore, scholars have investigated knowledge-sharing behaviours in various online communities (Cheung et al., 2013; Park et al., 2014; Song et al., 2010). Online Health Communities (OHCs) are one kind of an OC, where maintaining health information is a public concern. OHCs through social media and other web-based forums, facilitate their members to participate in health topics, even those with sensitive considerations such as pregnancy, menstruation, and sexuality (Fan et al., 2014; Rai et al., 2012).

OHCs have recently received substantial attention from health practitioners due to several considerations. Everyday users tend to be well-educated on disease causes, treatment advice and preventive actions by simply inputting personal health information into OHCs (Imlawi et al., 2020). Individuals go as far as to opt for self-diagnosing through OHCs rather than the traditional way by physically visiting hospitals (Tanis et al., 2016). Besides, OHCs grew impressively after observable internet technology advancement and emerged as a powerful medium among healthcare providers to be active members in OHCs (Zhao et al., 2013).

Participation in OHCs by healthcare workers can share their experiences, information, and feelings with each other and offer help and support (Fan et al.,

2014). One benefit of OHCs includes 24/7 access to information and assistance from individuals without any restrictions imposed by geographic location. The relatively free and less risk-oriented nature suggests that several opinions are always better for making decisions regarding health and medical concerns (Haynes, 2001; Lin et al., 2016; Mein et al., 2016). Previous studies show that OHCs are positively associated with a user's treatment options, health outlook, and outcomes (Zhang et al., 2017b). Participants who share knowledge within an OHC view the contribution as a perceived benefit as they may find happiness in enhancing their knowledge or social value in educating others (Liu et al., 2020; Zhang et al., 2017). Other perceived benefits may include financial incentives from the communities (such as fees or donations), the joy of interacting with other community members, and/or the increased reputation within the community due to their contributions (Yan et al., 2016).

The process of knowledge sharing is less effective within an organisation without the involvement and engagement of the human element (Ali et al., 2016). Several studies have identified the role of individual factors in knowledge-sharing behaviours (Abdel Fattah et al., 2020; Fullwood et al., 2019; Obrenovic et al., 2020). For instance, Shehab et al. (2018) reviewed 31 studies that investigated predictors of knowledge-sharing in different contexts; they found that the roles of individual factors are dominant. However, knowledge-sharing behaviours studies in Jordanian hospitals are scarce. As Alhalhouli (2014) reported, "Variables that enhance or dissuade knowledge sharing behaviours in the Jordanian hospitals have not been poorly recognised" (p. 919). Al-Dalaien et al. (2019) established a conceptual model of motivational factors of knowledge transfer in Jordanian hospitals. Aldohyan's et al. (2019) study in the Saudi context emphasised that "Hospitals should always refer to efficient knowledge sharing and educational strategies that render beneficial outcomes to patients, healthcare workers, and the public community" (p. 19).

Accordingly, the current study extends the previous literature and fills the gap by examining four individual factors (trust, reciprocity, reputation, and ability to share) with knowledge-sharing behaviours in OHCs. Additionally, this study examines the moderating effect of knowledge self-efficacy as it can change the strength of the direct effect between the above-mentioned individual factors and the knowledge-sharing behaviours of head nurses in Jordan.

This article is organised as follows. The first section discusses the study theories, hypotheses, and research model. This is followed by a section that presents the research methodology and analyses the results. Last, the implications and conclusions have been provided as well.

2. Literature review

2.1 Underpinning theory

Social Cognitive Theory (SCT) postulates that the mutually triangular interaction of individual factors like individual cognition, social factors such as social group (Online Community), environmental factors, and individual expectations and beliefs (Bandura, 1989, 2001) shape human behaviours. SCT primarily focuses on self-efficacy, considered as useful prescriptive and practical concepts formulated in modern psychology" (Betz, Klein, & Taylor, 1996). Other authors also provided their opinions on self-efficacy. For example, Lent (1996) states that self-efficacy refers to "people's judgment of their abilities to organize and implement courses of action required to achieve certain types of performance". The study moderator (i.e. self-efficacy) lays the foundation for personal achievement, personal well-being, and human motivation, human performance; Bandura (1977) assumed that people's level of motivation, emotional states, and actions depend more on what they believe than on what is objectively true. Self-efficacy reflects people's beliefs about their competence or effectiveness in carrying out tasks and tends to be more self-confident (Aslam et al., 2018).

Previous literature has also empirically confirmed this concept (Alireza et al., 2013; Chen et al., 2012; Tsai et al., 2010). SCT suggests that individual motivation and action are apparent bounded, and an individual is more or less likely to undertake a specified behaviour (Kreps et al., 2011; Font et al., 2016). Thus, the study model used social cognitive theory.

2.2 Hypotheses building

Over the last few decades, studies have emphasised the importance of individual self-efficacy and expectation in predicting individual health behaviours (e.g. Rgn & Rgn, 2002). Self-efficacy refers to people's judgment of their capabilities to organise and execute courses of action required to attain designated types of performance (Lent, 1996). It is "one of the most theoretically, heuristically and practically useful

concepts formulated in modern psychology" (Betz et al., 1996). Prior research has demonstrated that self-efficacy lays the foundation for personal achievements, personal well-being, and human motivation. Bandura (1977) explained, "People's level of motivation, affective states, and actions are based more on what they believe than on what is objectively true."

Knowledge self-efficacy is important in influencing the process of knowledge sharing and the influencing factors that contribute to knowledge sharing among online communities. For example, Hsieh et al.'s (2013) study showed that knowledge self-efficacy could moderate the relationships between reputation and pleasure in helping others share knowledge. Thus, the conclusion can be reached that self-efficacy strongly influences an individual's behaviour (Cherian & Jacob, 2013). Aligned with this, the current study investigates the moderating effect of self-efficacy on the relationship between individual factors and knowledge-sharing behaviour. The assumption is that when the level of knowledge and self-efficacy is high, head nurses are very confident in their ability to provide valuable knowledge.

Knowledge sharing in online communities has been given less attention to the relationship between knowledge self-efficacy and knowledge-sharing behaviour (Zhang et al., 2017). This may be an issue in knowledge sharing because complexity and knowledge barriers to exchanging knowledge among online communities may be seen as knowledge efficacy deficits (Lee et al., 2012; Memon et al., 2016).

Knowledge self-efficacy suggests that people who think their knowledge is valuable would be more likely to share greater knowledge (Shaari, Bakri, & Rahman, 2015). It is described as a function of self-beliefs with which individuals accomplish a particular work (Bandura, 1986), and knowledge self-efficacy can lead to greater productivity and performance. Knowledge self-efficacy is a type of self-assessment affecting decisions on how an individual will behave and be motivated under tasks and the level of effort asserted in the face of challenges.

Past researchers have linked knowledge self-efficacy to motivation and behaviour (Bandura, 1986; Igbaria et al., 1995; Nguyen et al., 2019). Those with higher levels of self-efficacy tend to perform better than those with lower levels (Zhao et al., 2005). Recently, researchers have concentrated on knowledge self-efficacy. This has been implemented in knowledge management to validate the effect of self-assessment, self-confidence, and motivation of individuals for knowledge sharing. Self-efficacy is highlighted as individual expectations of positive outcomes of behaviour since, if

individuals doubt the capability to complete the behaviour successfully, pursuing an action would be perceived as worthless. According to Wasko et al. (2005), an individual with high knowledge self-efficacy may feel happy answering questions easily, specifically questions from beginners. Consequently, such a person may develop a more positive behaviour towards sharing knowledge (Kankanhalli et al., 2005; Lai et al., 2013; Lin, 2007b). Additionally, their ethical commitment should strongly influence knowledge-sharing behaviour in online healthcare communities.

The current study anticipates that the influence of individual factors of trust, reciprocity, reputation, and ability to engage in knowledge-sharing behaviour will become stronger as head nurses gain more knowledge and self-efficacy in online healthcare communities (see Figure 1).

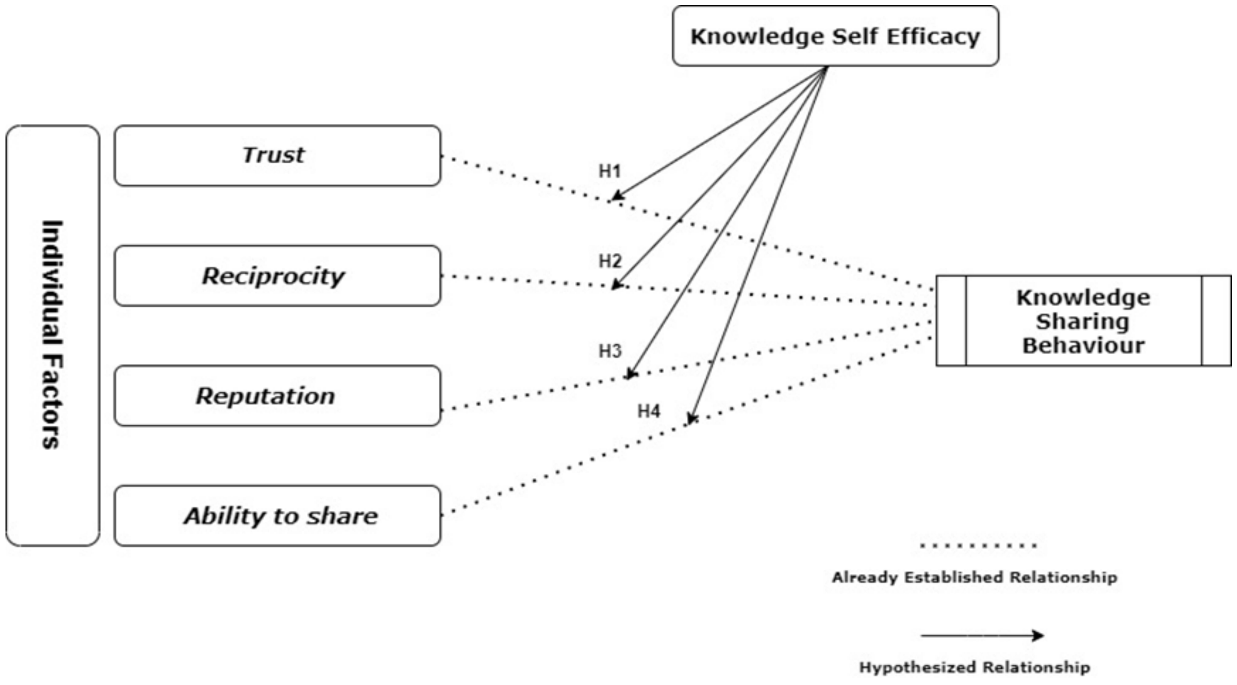

Figure 1. Research model

Accordingly, the following hypotheses are posited:

H1: Knowledge self-efficacy moderates the relationship between trust and knowledge-sharing behaviour.

H2: Knowledge self-efficacy moderates the relationship between reciprocity and knowledge-sharing behaviour.

H3: Knowledge self-efficacy moderates the relationship between reputation and knowledge-sharing behaviour.

H4: Knowledge self-efficacy moderates the relationship between the ability to share and knowledge-sharing behaviour.

3. Methodology

Why do you choose head nurses?

3.1 Design, Sampling, and Settings

A quantitative cross-sectional study was conducted using self-reported booklet surveys targeting individual head nurses. The population of this study was private hospitals in Jordan (n= 68). The researchers purposively selected the private hospitals in the capital (i.e. Amman) because these institutions have competitive advantages, technological capabilities, highest capacity, diversity in terms of speciality, supportive research cultures, and the highest number of hospitals located there (n= 32). The research team attained approval from 22 private hospitals with 322 head nurses, but 10 hospitals rejected participation in this study. The sample size was calculated based on the G*Power software package, which calculated that a minimum sample size of 74 was required with five independent variables, including the moderator. Accordingly, this study reached data from 283 respondents, which was satisfactory.

3.2 Ethical Considerations and Data Collection Procedures

Ethical approval number **UNITEN/COGS 23/2/1/PM20604** was attained from the *College of Graduate Studies, Universiti Tenaga Nasional*, Malaysia, on 25 April 2018. The hospitals approved their employees' voluntary participation in the study and encouraged their head nurses to participate. The first page of the booklet survey was a cover page that provided the study purposes, the necessary definitions, and the approval sign to conduct the study. *Written consent was obtained from all the respondents after they were informed regarding their right to withdraw from participation at any time, that data would be only for academic purposes, and their responses would be confidential.* Booklet surveys were distributed personally to all head nurses who were on duty during the data collection in 22 hospitals. 322 head nurses were invited to participate in this study. Accordingly, the data collection process started in May 2018 and lasted until October 2018. Of the 322 surveys distributed, the total usable surveys received were **283**, for an effective response rate of 84%.

The random sampling should be clearly stated: which random sampling method is applied?

Should be specified Validity and Reliability of questionnaire.

3.3 Measures

The study questionnaire was revised several times before starting the collection data process (i.e. content validity). The last version of the questionnaire includes two parts; the first asked demographic questions such as gender, age, education, internet usage, and experience. The second part included six scales (i.e., Trust, Reciprocity, Reputation, Ability to Share, Knowledge Self-efficacy, Knowledge Sharing Behaviour) and used a 5-point Likert ranging from 1 = strongly disagree to 5 = strongly agree. The first scale was *trust*, defined as employees' belief in good intent, competence, and reliability concerning contributing and reusing knowledge. The four items for the scale were adapted from Jarvenpaa and Leidner (1999) and Hassandoust et al. (2011). The second scale contained three items adapted from the *reciprocity* scale that Zhang et al. (2017a) modified, which referred to a belief that current sharing behaviour would cause future requests for knowledge to be easily satisfied by others. The third scale was *reputation*, which refers to a perception of improved reputation and image due to sharing knowledge in the online community. This was adapted from Kankanhalli et al. (2005). The fourth scale was the *ability to share*, which refers to the capabilities of conceiving and sharing meaning in different situations. The scale was adapted from Mohammadyari and Singh (2014). The fifth scale was *self-efficacy*, which means the degree of confidence in one’s ability to provide valuable knowledge to others. The scale was adapted from Aslam et al. (2018). The last scale was *knowledge sharing behaviour*, which refers to a process of knowledge exchange between individuals who disperse their obtained knowledge, experiences, and skills to others and groups (Zhang et al., 2017). The survey was written in the English language. Appendix 1 presents a list of items for each of the measures.

3.4 Data Analysis Techniques

This study used Smart PLS3 to test the hypotheses posited. Smart PLS3 uses a bootstrapping technique to estimate path coefficients and standard errors (Awang et al., 2015). Before running Smart PLS3, descriptive results were performed using SPSS Version 18.0.

4. Results

4.1 Demographics Characteristics

SPSS 18.0 descriptive and frequencies analysis output showed the 283 nurses' demographic characteristics. (See Table 1)

Table 1: Demographic Characteristics

Characteristic	Profile	N	%
Gender	Male	134	47.3
	Female	149	52.7
Age	25-30 years	86	30.4
	31-35 years	73	25.8
	36-40 years	58	20.5
	> 40 years	66	23.3
Education	Bachelor's	202	71.4
	High diploma	45	15.9
	Masters	33	11.7
	PhD	3	1.1
Internet usage	< 1hour	27	9.5
	1 - 3hours	149	52.7
	4 - 6 hours	66	23.3
	>6 hours	41	14.5
Experience	<5 years	17	6
	5-10 years	65	23
	>10 years	201	71
Total		283	

4.2 Validity and Reliability

Smart PLS 3.0 measurement or outer models detect if the collected data are valid and reliable. In this study, Convergent validity which (i.e. Cronbach's alpha, Composite reliability, and Average Variance Extracted) were tested and achieved acceptable value. Fornell-Larcker criterion and Heterotrait-Monotrait (HTMT) were examined by Smart PLS 3.0 measurement model and indicated to valid data (Hair et al., 2016). (See Table 2)

Table 2. Convergent & Discriminant Validity

	Convergent validity			*Discriminant validity (Fornell-Larcker criterion)						**Discriminant validity (HTMT Ratio)					
Constr.	α>.70	CR>.70	AVE>.50	TRU	REC	REP	ABS	KSB	KSE	TRU	REC	REP	ABS	KSB	KSE
TRU	.89	.92	.75	**.89**											
REC	.89	.93	.82	.19	**.90**					.84					
REP	.90	.93	.77	.72	.18	**.79**				.79	.82				
ABS	.92	.94	.81	.72	.26	.69	**.91**			.67	.72	.65			
KSE	.93	.95	.82	.57	.23	.61	.58	**.88**		.66	.77	.67	.63		
KSB	.78	.86	.61	.66	.34	.70	.29	.66	**.90**	.71	.62	.71	.52	.68	

Note: TRU: Trust, REC: Reciprocity, REP: Reputation, ABS: Ability to share, KSE: Knowledge Self-Efficacy, KSB: Knowledge Sharing Behaviour. α= Cronbach's alpha, CR = Composite reliability, AVE= Average variance extracted.
* Fornell-Larcker criterion: the values in **bold** are accepted if it > the corresponding row and column values.
** HTMT Ratio <.85 is valid.

Discriminant validity could also be performed by assessing the cross-loading of items (Hair et al., 2016). To achieve an acceptable level of cross loading, the indicators' (items) loading of the constructs should be higher than the loading on another construct, which was achieved as Table 3 shows.

Table 3. Cross loading of constructs

	ABS	KSB	KSE	REC	REP	TRU
ABS1	**0.910**	0.655	0.149	0.630	0.565	0.551
ABS2	**0.911**	0.643	0.129	0.590	0.488	0.543
ABS3	**0.915**	0.619	0.191	0.590	0.479	0.493
ABS4	**0.854**	0.654	0.195	0.754	0.519	0.571
KSB1	0.531	**0.688**	0.136	0.466	0.401	0.453
KSB3	0.575	**0.785**	0.161	0.585	0.504	0.508
KSB4	0.547	**0.806**	0.083	0.514	0.48	0.532
KSB5	0.584	**0.836**	0.18	0.583	0.513	0.518
KSE1	0.155	0.131	**0.882**	0.237	0.218	0.109
KSE2	0.202	0.184	**0.897**	0.258	0.238	0.149
KSE4	0.120	0.146	**0.906**	0.179	0.186	0.058
KSE5	0.180	0.179	**0.929**	0.257	0.210	0.122
REC1	0.673	0.623	0.256	**0.906**	0.560	0.570

REC2	0.627	0.640	0.240	**0.908**	0.513	0.567
REC3	0.649	0.617	0.213	**0.909**	0.509	0.554
REP1	0.453	0.468	0.179	0.46	**0.842**	0.471
REP2	0.559	0.575	0.245	0.536	**0.908**	0.520
REP3	0.508	0.539	0.185	0.537	**0.887**	0.518
REP4	0.483	0.553	0.216	0.500	**0.873**	0.480
TRU1	0.552	0.553	0.123	0.496	0.498	**0.850**
TRU2	0.537	0.584	0.130	0.554	0.462	**0.868**
TRU3	0.487	0.549	0.075	0.572	0.505	**0.883**
TRU4	0.509	0.547	0.101	0.528	0.502	**0.865**

4.3 Construct Cross-Validated Redundancy (Q^2)

The blindfolding output of SmartPLS is calculated to measure the predictive relevance of the latent variables of a study. Table 4 shows that Stone-Geisser Q^2 equal 1 – SSE/SSO. As a result of Henseler et al.'s (2009) procedures, a research model with $Q^2 > 0$ attained the accepted value of predictive relevance.

Table 4. *Construct cross-validated redundancy*

Latent variable	SSO	SSE	Q^2 =1- (SSE/SSO)
**Knowledge Sharing Behaviour**	1,632.00	1,112.16	0.318

Note: SSE is the sum of Squares of Prediction Errors; SSO is the Sum of Squares Observations.

4.4 Coefficient of Determination (R^2)

The output of the PLS3 structure produced a coefficient of determination values (R2) of knowledge sharing behaviour (KSB) as 0.695. This means that ABS, KSE, REC, REP, and TRU together explained 69.5% of knowledge-sharing behaviour among head nurses in Jordan. A larger R^2 value increases the predictive ability of the structural model. In the current study, R^2 is substantial according to Chin's (1998) classification of R^2 value.

4.5 Study Hypotheses Testing

This study investigated four hypotheses concerning the moderation effect of knowledge self-efficacy between trust, reputation, reciprocity, and ability to share with knowledge sharing behaviour. The result of 5000 bootstrap of 283 cases to measure the significance of the path coefficients with a 95% Confidence Interval

showed a moderation effect of knowledge self-efficacy in the relationship between trust, reputation, reciprocity, and knowledge-sharing behaviour (Table 5).

Table 5. Test of the Moderating Effect of Knowledge Self-Efficacy

#	Path	f^2	*β*	SE	T Value	*P* (Sig)
**H1**	TRU*KSE ---> KSB	0.026	0.149	0.069	2.166	0.03 **
**H2**	REC*KSE ---> KSB	0.032	-0.167	0.071	2.358	0.018 **
**H3**	REP*KSE---> KSB	0.078	0.192	0.053	3.640	0.001 ***
**H4**	ABS*KSE---> KSB	0.015	-0.119	0.066	1.794	0.073$^{(n.s)}$

Note: ***: p<0.01; **: p<0.05

In more detail, the moderating effect of knowledge self-efficacy (interaction between knowledge self-efficacy and trust, TRUST*KSE) exists in the relationship between reputation and knowledge-sharing behaviour. The results were also statistically significant (β =0.142, p=0.03) and positive, which revealed that knowledge self-efficacy was able to moderate the relationship between trust and knowledge-sharing behaviour positively. Based on these findings, trust was more positively effective on knowledge sharing behaviour when the knowledge self-efficacy is at a higher level; when the knowledge self-efficacy increases, this factor will increase; hence, trust will increase the knowledge sharing behaviour.

Concerning reciprocity, the results were statistically significant (β = -0.167, p=0.018). It was also negative effect; meaning that knowledge self-efficacy negatively moderated the relationship between reciprocity and knowledge-sharing behaviour. This finding indicated that, at a high level of knowledge self-efficacy, reciprocity had a lower effect on knowledge sharing behaviour and vice versa. In more detail, when the level of knowledge self-efficacy reduces, reciprocity would be more effective on knowledge sharing behaviour.

Reputation was also found to be a moderator and statistically significant and positive (β =0.192, p<0.001). This revealed that knowledge self-efficacy positively moderated the relationship between reputation and knowledge-sharing behaviour. Thus, it can be concluded that reputation was more positively effective on knowledge-sharing behaviour at a high level of knowledge self-efficacy. Likewise, if knowledge self-efficacy increases, the reputation factor will affect the level of knowledge-sharing behaviour.

Surprisingly, the bootstrapping calculation between the ability to share and sharing behaviour did not have a significant effect (β =-0.119, p=0.073). This means that knowledge self-efficacy did not moderate the relationship between the ability to share and knowledge-sharing behaviour.

5. Discussion

This is the first study investigating the knowledge-sharing behaviour of OCH in the Jordanian context. The main objective of this study was to assess the moderating effect of knowledge self-efficacy on the relationship between four individual factors and knowledge-sharing behaviour among head nurses in online healthcare communities in Jordan.

Lai and Hsieh (2013) found that reciprocity was a critical motivator of continued knowledge-sharing behaviour for people with low knowledge self-efficacy. First, they found that knowledge self-efficacy moderates trust and knowledge-sharing behaviour in online health care communities. If an individual has a strong sense of knowledge self-efficacy, he or she will have no problem sharing (Lai & Hsieh, 2013).

The current study found that knowledge self-efficacy among head nurses can increase the effect of trust on knowledge-sharing behaviour and higher knowledge self-efficacy. The effect of trust on the part of head nurses is more positive and effective regarding their knowledge-sharing behaviour. In line with Social Cognitive Theory, this finding suggests that nursing knowledge-sharing behaviour increases with their ability to control or behave.

Second, knowledge self-efficacy served as a moderator between reciprocity and knowledge-sharing behaviour; this finding was consistent with previous studies in different contexts (Aslam et al., 2018; Lai & Hsieh, 2013). More specifically, the moderating effect of knowledge self-efficacy between reciprocity and knowledge-sharing behaviour implies that an individual with low knowledge self-efficacy is more reciprocal in sharing knowledge than an individual with a high score of knowledge self-efficacy.

Third, the present study found that knowledge self-efficacy moderates reputation and knowledge-sharing behaviour. This implies that the effect of reputation on knowledge-sharing behaviour was high for the employee with a high level of self-efficacy. In other words, reputation strongly influenced knowledge contributors with high levels of self-knowledge efficacy (Lai et al., 2013; Nguyen et al., 2019). This

significance of the moderating role of knowledge sharing between reputation and knowledge sharing is also in line with social cognitive theory. As stated, the theory asserts that behaviour is the product of an individual's past experience and level of self-efficacy. Accordingly, knowledge self-efficacy increases the effectiveness of reputation in enhancing the knowledge-sharing behaviours among head nurses. Head nurses who gain reputations from online communities and have higher knowledge self-efficacy will be more likely to share knowledge in OHCs. The present study extends the understanding of the moderating role of knowledge self-efficacy between reputation and knowledge-sharing behaviour. It also extends the understanding of the applicability of knowledge self-efficacy among head nurses working in online health communities, specifically in Jordan.

The results contradicted the proposed hypothesis, as knowledge self-efficacy did not mediate between the ability to share and knowledge-sharing behaviour. This result might be due to inadequate knowledge-sharing activities at private hospitals, which may have shown that knowledge self-efficacy does not support their ability to share in OHCs. In addition, this result is consistent with Sitharthan et al. (2001) and Nguyen et al. (2019) studies that reported that self-efficacy does not always moderate the relationship between two personal variables.

5.1 Implications and Future Research

This study expanded the literature regarding knowledge-sharing behaviour, individual factors, and knowledge self-efficacy. Examining the study model in the healthcare sector in Jordan is not only considered to offer an extension of the literature. However, it also fills the gap in the existing literature by providing a comprehensive understanding of the above moderating effect of Knowledge Self-Efficacy, which could enrich knowledge-sharing behaviours.

The study's findings could benefit private hospitals in improving the knowledge-sharing behaviour of head nurses in OHCs by helping them understand essential factors that could affect their knowledge-sharing behaviour in these communities. Moreover, the findings would be helpful for Jordanian private hospitals in obtaining a better understanding of the main factors increasing the knowledge-sharing behaviour of head nurses. The role of individual factors includes trust, reciprocity, and reputation, which have been shown to improve knowledge sharing in online healthcare communities.

Furthermore, it provides practical contributions about the role of knowledge self-efficacy as a predictor of knowledge-sharing behaviour. Specifically, the results regarding the link between knowledge self-efficacy and knowledge-sharing behaviour offer clear insights for hospital management to avoid challenges affecting knowledge-sharing practices (Dammaj et al., 2016). The management can apply these findings in setting a plan for supporting knowledge self-efficacy. For example, head nurses should allocate specific time to share knowledge via online healthcare communities and connect sharing amounts to a "points system." The management could arrange workshops and seminars to increase the level of knowledge self-efficacy among head nurses and increase knowledge-sharing behaviour among its communities. Overall, this study gives top management at private hospitals more understanding of how knowledge self-efficacy can encourage head nurses to share their knowledge in online healthcare communities. Future research can explore new variables as independent, dependent, or moderating variables such as organisational and environmental factors or extend the investigation to more regions and sectors such as education and finance. Moreover, future research may investigate non-significant results in this study, such as the ability to share through the moderating effect of knowledge self-efficacy.

5.2 Study Limitations

This study has limitations. First, the generalizability of the current study's findings is limited in two aspects. In particular, the study involved one representative from among the head nurses of each department in the hospitals. However, other employees were not considered when making up the study sample. Second, the data collection was restricted to private hospitals in Amman city due to the ability to access data and other motivational reasons mentioned in the methodology section. Therefore, the findings may not be generalizable to other sectors in Jordan or other countries. Hence, comparable studies could be conducted in other sectors to consider more employees during the survey. Third, the study was completed in 2019; the data reported here were dated. However, because the study variables are interpersonal interactions, they are less likely to be affected by time (Jarrar et al., 2021). The last limitation of the study is that it involved only private hospitals in Amman. This could extend to other hospitals in different regions or other healthcare sectors in the future.

6. Conclusion

As technology and social media become advanced, knowledge-sharing behaviour is in OHCs to enhance the health status of individuals and communities. This study focused on knowledge self-efficacy and reflection on individuals' factors and knowledge-sharing behaviour. Head nurses with a high self-efficacy of knowledge can improve their knowledge-sharing behaviour. Knowledge self-efficacy moderates trust, reciprocity, and reputation with knowledge-sharing behaviour, while the ability to share did not. This study has several implications for private hospitals in Amman regarding the key roles of individual factors and knowledge self-efficacy in improving knowledge-sharing behaviour among head nurses in online healthcare communities. Accordingly, recognising the links explained by the study model could add value to the theory and practice.

Author Contributions: Conceptualisation, S.SH.; methodology, S.SH. and M.A.-B. and M.J.; software, A.M.; validation, H.M.K., M.D. and M.J.; formal analysis, S.SH.; investigation, M.A.-B.; resources, M.D. and B.K.D.; data curation, M.D., A.M. and M.J.; writing—original draft preparation, S.SH.; writing—review and editing, B.K.D., M.D., A.M., M.J., and M.A.-B.; visualisation, M.A.-B. and A.M.; project administration, M.J. All authors have read and agreed to the published version of the manuscript.

Funding: This research received no external funding.

Institutional Review Board Statement: This study obtained the ethical approval number UNITEN/COGS 23/2/1/PM20604 from the College of Graduate Studies, Universiti Tenaga Nasional - Malaysia, on 25 April 2018. This study is part of a PhD—thesis under the supervision of Universiti Tenaga Nasional, Malaysia.

Informed Consent Statement: Informed consent was obtained from all subjects involved in the study.

Data Availability Statement: The data presented in this study are available on request from the corresponding author.

Conflicts of Interest: The authors declare no conflict of interest.

References

Abdel Fattah, F. A. M., Mohamed, A. H. H. M., Bashir, M. I. A., & Al Alawi, A. M. M. (2020). Determinants of knowledge-sharing behaviour among students at higher educational institutions in Oman: A planned behaviour theoretical perspective of knowledge sharing. *Global Knowledge, Memory and Communication*, *70*(6–7), 611–636. https://doi.org/10.1108/GKMC-07-2020-0104

Al-Dalaien, A. H., Drus, S. M., & Kasim, H. (2019). A conceptual model of motivational factors of knowledge transfer for hospitals. International Journal of Engineering and Advanced Technology, *9*(1), 2313-2319. https://doi: 10.35940/ijeat.A2670.109119

Aldohyan, M., Al-Rawashdeh, N., Sakr, F. M., Rahman, S., Alfarhan, A. I., & Salam, M. (2019). The perceived effectiveness of MERS-CoV educational programs and knowledge transfer among primary healthcare workers: a cross-sectional survey. BMC infectious diseases, *19*(1), 1-9. https://doi.org/10.1186/s12879-019-3898-2

Alhalhouli, Z. T. (2014). Factors affecting knowledge sharing behavior among stakeholders in Jordanian hospitals using social networks. I*nternational Journal of Computer and Information Technology*, *3*(5), 919–928.

Ali, A. A., & Dominic, P. D. D. (2016). Organisational and individual factors impact on knowledge sharing practice: The association with cost reduction. *2016 3rd International Conference on Computer and Information Sciences (*pp. 536–541). https://doi.org/10.1109/ICCOINS.2016.7783272

Alireza, Bin Baba, M. S., Tamjid, H., & Gholipour, R. (2013). Information security - Professional perceptions of knowledge-sharing intention under self-efficacy, trust, reciprocity, and shared-language. *Computers and Education*, *68*, 223–232. https://doi.org/10.1016/j.compedu.2013.05.010

Armitage, C. J., & Conner, M. (1999). Predictive validity of the theory of planned behaviour: The role of questionnaire format and social desirability. *Journal of Community & Applied Social Psychology*, *9*(4), 261–272. https://doi.org/

10.1002/(SICI)1099-1298(199907/08)9:4%3C261::AID-CASP503%3E3.0.CO;2-5

Aslam, I., Safdar, T., & Shafiq, M. (2018). Does self-efficacy moderate the relationship between knowledge sharing and organisational citizenship behavior? (a quantitative research in civil secretariat Quetta). *European Online Journal of Natural and Social Sciences: Proceedings*, *7*(3), 232.

Awang, Z., Wan Afthanorhan, W. M. A., & Asri, M. A. M. (2015). Parametric and non parametric approach in Structural *European Online Journal of Natural and Social Sciences* Equation Modeling (SEM): The application of bootstrapping. *Modern Applied Science*, *9*(9), 58–67. https://doi.org/10.5539/mas.v9n9p58

Bandura, A. (1977). Self-efficacy: Toward a unifying theory of behavioral change. *Psychological Review*, *84*(2), 191–215. https://doi.org/10.1037/0033-295X.84.2.191

Bandura, A. (1986). Fearful expectations and avoidant actions as coeffects of perceived self-inefficacy. *American Psychologist, 41*(12), 1389–1391. https://doi.org/10.1037/0003-066X.41.12.1389

Bandura, A. (1989). Human agency in social cognitive theory. *American Psychologist*, *44*(9), 1175-1184. https://psycnet.apa.org/doi/10.1037/0003-066X.44.9.1175

Bandura, A. (1999). Social cognitive theory of personality. In L. Pervin & O. John (Ed.), *Handbook of personality* (2nd ed., pp. 154-196). Guilford Publications.

Bandura, A. (2001). Social cognitive theory: An agentic perspective. *Annual Review of Psychology*, *52*(1), 1–26. https://doi.org/10.1111/1467-839X.00024

Betz, N. E., Klein, K. L., & Taylor, K. M. (1996). Evaluation of a short form of the career decision-making self-efficacy scale. *Journal of Career Assessment*, *4*(1), 47–57. https://doi.org/10.1177%2F106907279600400103

Bock, G. W., & Kim, Y. G. (2002). Breaking the myths of rewards: An exploratory study of attitudes about knowledge sharing. *Information Resources Management Journal (IRMJ)*, *15*(2), 14-21. 10.4018/irmj.2002040102

Bouma, S. (2011). *Smarter Knowledge Sharing Quantitative research into the perceived usefulness of Lotus Live as knowledge sharing system" by Dutch healthcare professionals* (Master's Thesis, Tilburg University). http://arno. uvt.nl/show.cgi

Burke, J. A., Estrin, D., Hansen, M., Parker, A., Ramanathan, N., Reddy, S., & Srivastava, M. B. (2006). *Participatory sensing*. UCLA: Center for Embedded Network Sensing.

Chen, S., Chuang, Y., & Chen, P. (2012). Knowledge-based systems behavioral intention formation in knowledge sharing: Examining the roles of KMS quality, KMS self-efficacy, and organisational climate. *Knowledge-Based Systems*, *31*, 106–118. https://doi.org/10.1016/j.knosys.2012.02.001

Cherian, J., & Jacob, J. (2013). Impact of self efficacy on motivation and performance of employees. *International Journal of Business and Management*, *8*(14), 80–88. https://doi.org/10.5539/ijbm.v8n14p80

Cheung, C. M. K., Lee, M. K. O., & Lee, Z. W. Y. (2013). Understanding the continuance intention of knowledge sharing in online communities of practice through the post- knowledge- sharing evaluation processes. *Journal of the Association for Information Science and Technology*, *64*(7), 1357–1374.

Chin, W. W. (1998). Issues and opinion on structural equation modeling. *MIS Quarterly*, *22*(1), 7-16. http://www.jstor.org/stable/249674

Dammaj, A., Alawneh, A., & Sweis, R. J. (2016). Investigating the relationship between knowledge sharing and service quality in private hospitals in Jordan Ayman Abu Hammad. *Productivity and Quality Management*, *17*(4), 437–455.

Fan, H., Lederman, R., Smith, S. P., & Chang, S. (2014). How trust is formed in online health communities: A process perspective. *Communications of the Association for Information Systems*, *34*(1), 531–560.

Font, X., Garay, L., & Jones, S. (2016). Annals of Tourism Research A Social Cognitive Theory of sustainability empathy. Annals of Tourism Research, *58*, 65–80. https://doi.org/10.1016/j.annals.2016.02.004

Fullwood, R., Rowley, J., & McLean, J. (2019). Exploring the factors that influence knowledge sharing between academics. *Journal of Further and Higher Education*, *43*(8), 1051–1063. https://doi.org/10.1080/0309877X.2018.1448928

Garson, G. D. (2016). *Partial least squares: Regression & structural equation models*. Statistical Associates Publisher.

Hair, J. F., Hult, G. T. M., Ringle, C. M., & Sarstedt, M. (2016). *A primer on partial least squares structural equation modeling (PLS-SEM)* (2nd ed.). SAGE

Publishing.

Hassandoust, F., Logeswaran, R., & Farzaneh Kazerouni, M. (2011). Behavioral factors influencing virtual knowledge sharing: Theory of reasoned action. *Journal of Applied Research in Higher Education*, *3*(2), 116–134. https://doi.org/10.1108/17581181111198665

Henseler, J., Christian M. Ringle, & Sinkovics, R. R. (2009). The use of partial least squares path modeling in international marketing. In G. David Garison (Ed.), *New challenges to international marketing* (pp. 277–319). Emerald Group Publishing Limited.

Hsieh, P. J., Lai, H. M., & Kuo, P. Y. (2013). Physician acceptance behavior of the electronic medical records exchange: An extended decomposed theory of planned behavior. *Proceedings - Pacific Asia Conference on Information Systems, PACIS 2013*. https://aisel.aisnet.org/pacis2013/197/

Hsu, M., Ju, T. L., Yen, C., & Chang, C. (2007). Knowledge sharing behavior in virtual communities: The relationship between trust, self-efficacy, and outcome expectations, *65*, 153–169. https://doi.org/10.1016/j.ijhcs.2006.09.003

Imlawi, J., & Gregg, D. (2020). Understanding the satisfaction and continuance intention of knowledge contribution by health professionals in online health communities. *Informatics for Health and Social Care*, *45*(2), 151-167. https://doi.org/10.1080/17538157.2019.1625053

Igbaria, M., & Iivari, J. (1995). The effects of self-efficacy on computer usage. *Omega*, *23*(6), 587–605. https://doi.org/10.1016/0305-0483(95)00035-6

Jarrar, M.; Al-Bsheish, M.; Aldhmadi, B.K.; Albaker, W.; Meri, A.; Dauwed, M.; Minai, M.S. (2021). Effect of practice environment on nurse reported quality and patient safety: The mediation role of person-centeredness. *Healthcare*, *9*, 1578. doi: 10.3390/healthcare9111578

Jinyang, L. (2015). Knowledge sharing in virtual communities: A social exchange theory perspective. *Journal of Industrial Engineering and Management*, *8*(1), 170–183. https://doi:10.3926/jiem.1389

Kankanhalli, Tan, & Wei. (2005). Contributing knowledge to electronic knowledge repositories: An empirical investigation. *MIS Quarterly*, *29*(1), 113. https://doi.org/10.2307/25148670

Khan, G. F., Sarstedt, M., Shiau, W.-L., Jr., J. F. H., Ringle, C. M., & Fritze, M.

(2019). Methodological research on partial least squares structural equation modeling (PLS-SEM): An analysis based on social network approaches. *Internet Research, Forthcoming*, *29*(3), 407-429. https://doi.org/10.1108/IntR-12-2017-0509

Kim, Y.-M., Newby-Bennett, D., & Song, H.-J. (2012). Knowledge sharing and institutionalism in the healthcare industry. *Journal of Knowledge Management*, *16*(3), 480–494.

Lai, H., & Hsieh, P.-J. (2013). The decision to continue sharing knowledge in virtual communities: The moderating role of knowledge-sharing experience and knowledge self-efficacy. In *PACIS 2013 Proceedings* (pp. 6–18).

Lee, C. S., & Ma, L. (2012). News sharing in social media: The effect of gratifications and prior experience. *Computers in Human Behavior*, *28*(2), 331–339.

Lent, R. W. (1996). Career development from a social cognitive perspective. In D. Brown & L. Brooks (Eds.), *Career choice and development* (pp. 373-421). Jossey-Bass.

Lin, H.-F. (2007a). Effects of extrinsic and intrinsic motivation on employee knowledge sharing intentions. *Journal of Information Science*, *33*(2), 135–149. ttps://doi.org/10.1177/0165551506068174

Lin, H.-F. (2007b). Knowledge sharing and firm innovation capability: an empirical study. *International Journal of Manpower*, *28*(3/4), 315–332. https://doi.org/10.1108/01437720710755272

Lin, T. C., Lai, M. C., & Yang, S. W. (2016). Factors influencing physicians' knowledge sharing on web medical forums. *Health Informatics Journal*, *22*(3), 594–607.

Liu, W., Fan, X., Ji, R., & Jiang, Y. (2020). Perceived community support, users' interactions, and value co-creation in online health community: The moderating effect of social exclusion. *International Journal of Environmental Research and Public Health*, *17*(1), 204. https://doi.org/10.3390/ijerph17010204

Lu, L., Leung, K., & Koch, P. T. (2006). Managerial knowledge sharing: The role of individual, interpersonal, and organisational factors. *Management and Organization Review*, 2(1), 15 - 41. doi: https://doi.org/10.1111/j.1740-8784.2006.00029.x

Mein, J., Gao, G., & Agarwal, R. (2016). The creation of social value: Can an online

health community reduce rural-urban health disparities? *MIS Quarterly*, *40*(1).

Memon, M. A., Nor, K. M., & Salleh, R. (2016). Personality traits influencing knowledge sharing in student-supervisor relationship: A structural equation modelling analysis. *Journal of Information & Knowledge Management*, *15*(2), 1650015. https://doi.org/10.1142/S0219649216500155

Mc Evoy, P. J., Ragab, M. A., & Arisha, A. (2019). The effectiveness of knowledge management in the public sector. Knowledge Management Research & Practice, 17(1), 39-51. https://doi.org/10.1080/14778238.2018.1538670

Mohammadyari, S., & Singh, H. (2014). SC. *Computers & Education*. *82*, 11-15. https://doi.org/10.1016/j.compedu.2014.10.025

Mozaffar, Hajar, and Niki Panteli. The online community knowledge flows: Distance and direction." *European Journal of Information Systems* 31.2 (2022): 227-240.

Mura, M., Mura, M., Lettieri, E., Lettieri, E., Radaelli, G., Radaelli, G., … Spiller, N. (2016). Behavioural operations in healthcare: A knowledge sharing perspective. *International Journal of Operations & Production Management*, *36*(10), 1222–1246.

Nguyen, T. M., Siri, N. S., & Malik, A. (2021). Multilevel influences on individual knowledge sharing behaviours: the moderating effects of knowledge sharing opportunity and collectivism. *Journal of Knowledge Management*, *26*(1), 70-87.

Nguyen, T. M., Nham, T. P., Froese, F. J., & Malik, A. (2019). Motivation and knowledge sharing: a meta-analysis of main and moderating effects. Journal of Knowledge Management.

Obrenovic, B., Jianguo, D., Tsoy, D., Obrenovic, S., Khan, M. A. S., & Anwar, F. (2020). The enjoyment of knowledge sharing: Impact of altruism on tacit knowledge-sharing behavior. *Frontiers in Psychology*, *11*(July), 1–16. https://doi.org/10.3389/fpsyg.2020.01496

Park, J. H., Gu, B., Leung, A. C. M., & Konana, P. (2014). An investigation of information sharing and seeking behaviors in online investment communities. *Computers in Human Behavior*, *31*, 1–12.

Pi, S.-M., Chou, C.-H., & Liao, H.-L. (2013). A study of Facebook Groups members' knowledge sharing. *Computers in Human Behavior*, *29*(5), 1971–1979.

Radaelli, G., Lettieri, E., Mura, M., & Spiller, N. (2014). Knowledge sharing and innovative work behaviour in healthcare: A micro-level investigation of direct and indirect effects. *Creativity and Innovation Management*, *23*(4), 400–414.

Rai, P., Kumar, P. S., Oh, S., Kwon, H., Mathur, G. N., Varadan, V. K., & Agarwal, M. P. (2012). Smart healthcare textile sensor system for unhindered-pervasive health monitoring. In *Nanosensors, Biosensors, and Info-Tech Sensors and Systems 2012* (Vol. 8344, p. 83440E). International Society for Optics and Photonics.

Rgn, A. H., & Rgn, H. E. W. (2002). Role of self- efficacy and behaviour change. International journal of nursing practice, 8(2), 106-115. https://doi.org/10.1046/j.1440-172x.2002.00352.x

Shaari, R., Bakri, N., & Rahman, A. A. (2015). Antecedents of knowledge sharing behavior among nurses: Towards research agenda. *Procedia - Social and Behavioral Sciences*, *171*, 635–641. https://doi.org/10.1016/j.sbspro.2015.01.171

Shehab, S., Rahim, R. E. A., & Daud, S. (2018). A review of individual factors on knowledge sharing: Evidence from the empirical literature. *International Journal of Engineering and Technology (UAE)*, *7*(4), 186–194. https://doi.org/10.14419/ ijet.v7i4.15.21444

Sitharthan, G., Hough, M. J., Sitharthan, T., & Kavanagh, D. J. (2001). The alcohol helplessness scale and its prediction of depression among problem drinkers. *Journal of Clinical Psychology*, *57*(12), 1445–1457.

Song, P., Zhang, C., Xu, Y. C., & Huang, L. (2010). Brand extension of online technology products: Evidence from search engine to virtual communities and online news. *Decision Support Systems*, *49*(1), 91–99.

Tanis, M., Hartmann, T., & te Poel, F. (2016). Online health anxiety and consultation satisfaction: A quantitative exploratory study on their relations. *Patient Education and Counseling*, *99*(7), 1227–1232.

Tsai, M.-T., & Cheng, N.-C. (2010). Programmer perceptions of knowledge-sharing behavior under social cognitive theory. *Expert Systems with Applications*, *37*(12), 8479–8485.

Tran, T. K. P. (2021). The effect of knowledge sharing and innovativeness on organisational performance: An empirical study in Vietnam. *The Journal of*

Asian Finance, Economics and Business, *8*(8), 503-511.

Wasko, M., & Faraj, S. (2005). Why should I share? Examining social capital and knowledge contribution in electronic networks of practice. *MIS Quarterly*, *29*(1), 35–57.

Yan, Z., Wang, T., Chen, Y., & Zhang, H. (2016). Knowledge sharing in online health communities: A Social Exchange Theory perspective. *Information & Management*, *53*(5), 643–653. https://doi.org/10.1016/j.im.2016.02.001

Zhang, X., Liu, S., Chen, X., Zhang, X., Liu, S., & Chen, X. (2017a). Social capital, motivations, and knowledge sharing intention in health Q&A communities. *Management Decision*, *55*(7), 1536–1557.

Zhang, X., Liu, S., Deng, Z., & Chen, X. (2017b). Knowledge sharing motivations in online health communities: A comparative study of health professionals and normal users. *Computers in Human Behavior*, *75*, 797–810. https://doi.org/10.1016/j.chb.2017.06.028

Zhao, H., Seibert, S. E., & Hills, G. E. (2005). The mediating role of self-efficacy in the development of entrepreneurial intentions. *Journal of Applied Psychology*, *90*(6), 1265-1272.

Zhao, J., Ha, S., & Widdows, R. (2013). Building trusting relationships in online health communities. *Cyberpsychology, Behavior, and Social Networking*, *16*(9), 650–657.

Appendix 1

Scale	Code	Statement	Source(s)
**Trust**	TRU1	We were usually considerate of one another's feelings in online healthcare communities.	Jarvenpaa and Leidner (1999)
	TRU2	The people in online healthcare communities were friendly.	Hassandoust et al., (2011)
	TRU3	I could rely on those with whom I worked in online healthcare communities.	
	TRU4	Overall, the people in online health communities were trustworthy.	
**Reciprocity**	REC1	When I share knowledge in online healthcare communities, I believe that my questions will be answered in the future.	Bock et al. (2005)
	REC2	I believe that other members whom I interact with would help me whenever I am in need in online healthcare communities.	Zhang et al. (2017a)
	REC3	When I share my knowledge in online healthcare communities, I expect the other members to respond whenever I am in need.	
**Reputation Scale**	REP1	I earn respect from others by participating in online healthcare communities.	Wasko and Faraj (2005)
	REP2	I feel that participation improves my status in online healthcare communities.	Zhang et al. (2017a)
	REP3	My participation in online healthcare communities can enhance my reputation in my professional field.	
	REP4	I can earn feedback or rewards through participation by representing my reputation and status in the online healthcare communities.	
**Ability to Share**	ABS1	I am fully capable of sharing my knowledge with others in online healthcare communities.	Armitage et al. (1999)
	ABS2	If it depended only on me, I would exhaustively share my knowledge in online healthcare communities.	Radaelli et al.(2014)
	ABS3	I am fully capable of articulating my knowledge in written or spoken form in online healthcare communities.	
	ABS4	I believe I am fully capable of sharing my	

		knowledge at any time in online healthcare communities.	
	ABS5	The knowledge I share with my colleagues would be very useful to them in online healthcare communities.	
**Knowledge Self-efficacy**	KSE1	My personal expertise will display its value if shared within the online healthcare communities.	Bock and Kim (2002)
	KSE2	My limited knowledge, even if shared, will generate little effect within the online healthcare communities.	Lu et al. (2006)
	KSE3	I am confident that my knowledge sharing would improve work processes in the online healthcare communities.	
	KSE4	I am confident that my knowledge sharing would increase the productivity in the online healthcare communities	
**Knowledge Sharing Behaviour**	KSB1	In daily work, I take the initiative to share my work-related knowledge with my colleagues in online healthcare communities.	Bock and Kim (2001)
	KSB2	I keep my work experience and never share it with others easily in online healthcare communities.	Lu et al. (2006)
	KSB3	After learning new knowledge useful to work, I promote it to let more people learn it in online healthcare communities.	
	KSB4	I actively use online healthcare community sources available to share my knowledge.	
	KSB5	So long as the other colleagues need it, I always tell whatever I know without any hoarding in online healthcare communities	

Click here to access/download
Supporting Information
Knowledge Sharing Behaviour, first round revesion.docx

Track changes, first round

Click here to access/download
Supporting Information
changes, second reound revision.docx

Track changes, first round

Click here to access/download

Supporting Information

track changes, first round version.docx
