# Supplementary material for: Knowledge sharing behaviour among head nurses in online health communities: The moderating role of knowledge self-efficacy
Source: PLoS One. 2023 Jan 19;18(1):e0278721. doi: 10.1371/journal.pone.0278721 (PMC9851523; doi:10.1371/journal.pone.0278721)
Supplement: Attachment — Submitted filename: Response to Reviewers.docx [file pone.0278721.s004.docx]

Academic Editor of PLOS ONE

Journal Requirements:
Please review your reference list to ensure that it is complete and correct. If you have cited papers that have been retracted, please include the rationale for doing so in the manuscript text, or remove these references and replace them with relevant current references. Any changes to the reference list should be mentioned in the rebuttal letter that accompanies your revised manuscript. If you need to cite a retracted article, indicate the article’s retracted status in the References list and also include a citation and full reference for the retraction notice.

Thank you for the comment.

References and study format has revised accordingly, and all unrelated references were removed accordingly.

Reviewer #1:

1- **The social cognitive theory that is found since 1996, that might be questionable this theory still suitable for using at the present. The authors might illustrate the evidences support.**

Thank you for the comment.

The social cognitive theory (SCT) is frequently used to guide behavior change interventions such as Knowledge Sharing Behaviour. It may be particularly useful for examining how individuals interact with their surroundings. The SCT can be used to understand the influence of social determinants of health and a person's experiences on behavior change. Comparing to other theories in this regard such as social exchange theory was developed in 1958, SCT is still current and many recent articles were used this theory.

for example:

Lin, H. C., & Chang, C. M. (2018). What motivates health information exchange in social media? The roles of the social cognitive theory and perceived interactivity. *Information & Management*, *55*(6), 771-780.‏

Ahmed, Y. A., Ahmad, M. N., Ahmad, N., & Zakaria, N. H. (2019). Social media for knowledge-sharing: A systematic literature review. *Telematics and informatics*, *37*, 72-112.‏

Lin, X., & Kishore, R. (2021). Social media-enabled healthcare: a conceptual model of social media affordances, online social support, and health behaviors and outcomes. *Technological Forecasting and Social Change*, *166*, 120574.‏

2- **It not necessary shows the hypotheses setting process of this study. That might be shows in review literature part.**

Thank you. Hypotheses testing and study framework was established in the literature review part

3- **Due to using the part analysis. This statistic is the family of multivariate statistic. The authors should choose another method for calculate minimum sampling size.**

Thank you for this comment.

It was calculated by using another method and added to the manuscript accordingly. “Beside G*Power software package, Krejcie and Morgan, (1970) sample size formula was used to get minimum number of respondents to be surveyed, which is 210”.

4- **There are some measure are established since 1999 the authors should examine the psychometric properties before using collect data.**

The reviewer means “Jarvenpaa, S. L., & Leidner, D. E. (1999). Communication and trust in global virtual teams. *Organization science, 10*(6), 791-815”

Despite this paper being old to some extent, however, it strong and citable one (more than 4000 citations). Moreover, the psychometric properties of this measure such as validity and reliability were examined in this study and it was valid and reliable.

5- **In this study has found the lower Beta values (see in table 5), the authors should explain the reasons and evidences support why the knowledge self-efficacy is not mediate between the ability.**

Yes, the study empirical results show no moderation effect of knowledge self-efficacy between the ability and knowledge-sharing behaviors.

Justification in the text is “This result might be due to inadequate knowledge-sharing activities at private hospitals, which may have shown that knowledge self-efficacy does not support their ability to share in OHCs. In addition, this result is consistent with Sitharthan et al. (65) and Nguyen et al. (3) studies that reported that self-efficacy does not always moderate the relationship between two personal variables”

Reviewer #2:

**The authors have made complete revisions to the paper to the satisfaction of all recommendations. The main strength of this paper is its strong literary references. However, the authors may also modify the Abstract composition to be a single paragraph without any subheading in this section**

Thank you for this comment. Abstract become a single paragraph without any subheading

Reviewer #3:

**The manuscript revision and response by author, Knowledge Sharing Behaviour among Head Nurses in Online Health Communities: The Moderating Role of Knowledge Self-Efficacy. I think you improve all comments that I reviewed this manuscript. Please recheck all references.**

Thank you for your reviewing our work. References and study format has revised accordingly, and all unrelated references were removed accordingly.

Reviewer #4:

**Knowledge Sharing Behaviour among Head Nurses in Online Health Communities: The Moderating Role of Knowledge Self-Efficacy. Thank you for your work hard to revise manuscript. I follow your response referees. I think Its improve to valuable article to publish**.

Thank you for your reviewing our work

We confirm that this work is non-funded and original and has not been published anywhere, nor is it currently under consideration for publication elsewhere.

Note: The data uploaded in the system to confirm the availability for future researchers requesting access to the data from corresponding authors.

Please address all correspondence concerning this manuscript to me at [mutaman.jarrar@yahoo.com, [mkjarrar@iau.edu.sa](mailto:mkjarrar@iau.edu.sa)].

Thank you for your consideration of this manuscript.

Sincerely,

Mu’taman Jarrar
